# News and Coming Events

**Published:** 1909-01-16

**Authors:** 


### News and Coming Events.

Mr. G. N. Biggs, F.R.C.S., has been appointed Aural Surgeon to the Evelina Hospital for Sick Children.

Mr. H. H. Clutton, M.C., F.R.C.S., has been appointed by His Majesty the King to be consulting surgeon to the Osborne Convalescent Hospital for Officers.

The Surrey County Council has appointed Mr. Edward Hinks public analyst for the county in succession to the late Sir Thomas Stevenson, M.D., to whom he was senior assistant for seven years.

The Queen has given a donation of £1,000 to be expended in the purchase of extra-regulation articles which will add to the comfort and convenience of sick soldiers in military hospitals at home stations nursed by Queen Alexandra's Imperial Military Nursing Service.

Dr. Charles Augustus West, a well-known Cornish magistrate, died suddenly on the evening of January 11 at Bodmin. Dr. West was eighty-one years of age. He qualified M.R.C.S.(Eng.) and L.S.A.(Lond.) in 1849, and took the degree of M.D. (St. Andrew's) in 1862.

The United States Ambassador in Berlin, on behalf of Mr. Carnegie, has handed to the bankers of the Robert Koch Fund for the campaign against tuberculosis a sum of £25,000. This represents the amount of Mr. Carnegie's promised contribution to the fund, which now has a capital of £57,500.

A public subscription list has been opened in the Isle of Wight with the object of providing a county public memorial of the late Dr. J. Groves, J.P., who was for many years medical officer of the rural district of the Isle of Wight, and was actively associated with various public institutions in the island—philanthropic, literary, sporting and horticultural. Dr. Groves was President of the Society of Medical Officers of Health during session 1903-4.

Mr. John Charles Lane, of Moseley, Worcestershire, solicitor, bequeathed £100 each to the Birmingham General Hospital, the Queen's Hospital, Birmingham, the Birmingham Blind Asylum, the Birmingham Deaf and Dumb Institute, the Birmingham Ear and Throat Hospital, the Birmingham Eye Hospital, the Birmingham Children's Hospital, the Jaffray Hospital, the Birmingham Skin and Lock Hospital, the Middlemore Homes, and the Moseley Park Hospital. After family bequests have been satisfied the ultimate residue of his property, which has been valued at £31,280, is to be divided equally between the above charitable institutions, which will apparently share between them a further £12,000.

The students of the Faculty of Medicine of the University of Paris on January 7 sent a deputation to the Ministry of Education to plead for the remission of the sentence of exclusion from all lectures until March 1, which has been passed upon medical students of the first and second years in consequence of last month's riotous protests against the new qualifying certificate for medical degrees. At a Cabinet Council held on January 11, the Minister of Public Instruction said that he was cancelling the recent examination for the admission to fellowships of the Faculty of Medicine, which had aroused such fierce opposition from students; and he added that he was authorising the reopening of the Faculty for students of the first and second year.

Mr. Norman Patterson, M.B., B.Ch.Edin., F.R.C.S. Eng., has been appointed assistant surgeon to the Golden Square Hospital for Diseases of the Throat.

The annual dinner of the West London Medico-Chirurgical Society will take place at the Great Central Hotel, Marylebone, on Friday, February 12.

Surgeon-General Gerald Bomford, C.I.E., M.D., Director-General of the Indian Medical Service, has been created a Knight Commander of the Indian Empire.

The Lord Chancellor has placed on the Commission of the Peace for the County of London the name of Surgeon-General Sir Charles Cuffe, K.C.B., Army Medical Staff (retired).

An International Hygienic Exhibition is being organised at Rio de Janeiro this year in connection with the Latin American Congress in that city. The exhibition will be open from August 1 until September 30.

The Kensington General Hospital, which until some two years ago was known as the Queen's Jubilee Hospital, Earl's Court, is about to apply to the Board of Trade for permission to change its name to Kensington and Fulham General Hospital.

The Lord President of the Council has appointed Mr. Francis E. Fremantle, M.A., M.B., M.R.C.P., F.R.C.S., D.P.H., medical officer of health for the county of Hertford, to be a member of the committee appointed to consider the working of the Midwives Act, 1902. Mr. Fremantle has also been appointed to the Edward Jenner Lectureship in Public Health at St. George's Hospital Medical School.

A provincial sessional meeting of the Royal Sanitary Institute will be held in Manchester on Friday, January 22, including, at 2.30 p.m., a visit to the new Manchester Royal Infirmary, and at 7 p.m. a discussion in the Municipal School of Technology on the Manchester Royal Infirmary from the Hygienic, Sanitary, and Æsthetic Points of View, which will be opened by Mr. Edwin T. Hall, Vice-President R.I.B.A., followed by Mr. John Brooke, F.R.I.B.A. (architects of the infirmary). The chair will be taken by Mr. H. D. Searles Wood, F.R.I.B.A. Tickets for admission of visitors may be had on application to Professor J. Radcliffe, Municipal School of Technology, Manchester, and of the institute secretary, Mr. E. White Wallis.

In the Divorce Division on January 12, the President (Sir Gorell Barnes) pointed out to the witnesses how, under the Oaths Act, they could, if they chose, be sworn in the Scottish form, than which nothing could be more emphatic or more suitable. He added that notices drawing attention to this manner of being sworn had been conspicuously posted for several years in both Divorce Courts, but it was a surprising fact that practically nobody except a few Scottish doctors had taken advantage of the opportunity afforded them. In the Divorce Courts Testaments specially bound with material which permitted cleansing had been provided, so that the oath taken in the ordinary way by kissing the Book was made as little objectionable as possible. His Lordship has further directed that in addition to the notices exhibited with regard to the taking of the Scottish oath the officers swearing the witnesses shall in future ask persons in every instance if they desire to be sworn in the Scottish form or not.

